# Supplementary material for: Novel anthropometric indices and cognitive function among iranian older adults: Bushehr Elderly Health (BEH) program
Source: PLoS One. 2026 Apr 30;21(4):e0343425. doi: 10.1371/journal.pone.0343425 (PMC13132206; doi:10.1371/journal.pone.0343425)
Supplement: Attachment — Submitted filename: Response to Reviewers.docx [file pone.0343425.s002.docx]

**Dear editorial manager and Reviewers:**

Thank you for providing comments on our manuscript entitled “**Novel Anthropometric Indices and Cognitive Function among Iranian Older Adults: Bushehr Elderly Health (BEH).** Those comments are all valuable and very helpful for revising and improving our manuscript, as well as providing important guidance to our research.

**PLOS ONE style requirements**: We have fully reformatted the manuscript (main text, title page, figures, and tables) according to the official PLOS ONE templates provided in the links.

**Data Availability:**

The data used in this study are from the Bushehr Elderly Health (BEH) program, a population-based cohort study owned and governed by the Ministry of Health and Medical Education of the Islamic Republic of Iran through the Persian Cohort and Biobank regulations. Due to ethical and legal restrictions on sharing potentially identifiable human data and national regulations, the raw dataset cannot be made publicly available. However, the de-identified minimal dataset underlying the results of this study is available upon reasonable request to the corresponding author.

This statement is also added to the manuscript.

**Regards**

**Authors**

Reviewer 1:

| **Comments** | **Responses** | **Line number** |
| --- | --- | --- |
| **1. Some sentences are long and could be broken into shorter sentences for readability.** | Thank you. We have carefully checked the entire manuscript and edited long sentences into shorter ones where possible to improve readability. Please let us know if you identify any remaining areas that need further adjustment. | Whole manuscript |
| **2. References to prevalence in Iran could include more recent statistics if available.** | We have added more recent statistics on prevalence of cognitive impairment in Iran, including references 4–6, to ensure the data are up to date. | References 4-6 |
| **3. Consider emphasizing why these indices are practical alternatives to HOMA-IR (cost and accessibility) a bit earlier to strengthen the rationale.** | We have emphasized earlier that these indices are practical alternatives to HOMA-IR due to their lower cost and greater accessibility. | Lines 87-89 |
| **4. Consider summarizing borderline associations separately from statistically significant results to improve clarity.** | Thanks. We decided to remove some of non-significant associations from the text. | Lines 252-255 |
| **5. Some sentences are repetitive (e.g., noting no association for TyG and LAP in multiple places). Consolidating these points could improve readability.** | Thank you for the comment. We have carefully reviewed the manuscript and edited the repetitive sentences in the Discussion and Conclusion sections to improve readability. | Lines 273-275  Lines 302-310 Lines 371-374 |
| 6. **Clarify whether quartile-based analyses were adjusted for multiple comparisons.** | Thank you for the comment.  To examine this relationship, we conducted quantitative and qualitative analysis, following the precedent of similar studies (https://doi.org/10.1186/s12944-023-01959-0). |  |

| **Comments**  Reviewer 2: | **Responses** | **Line number** |
| --- | --- | --- |
| **The study design is cross-sectional, yet several statements imply causality (e.g., “interplay between metabolic indices and cognitive function”). These should be rephrased to reflect association, not cause–effect.** | We have reviewed the manuscript and revised the relevant statements to emphasize **association** rather than causation or “interplay,” ensuring alignment with the cross-sectional design. If any phrasing still appears causal, please let us know and we will adjust it further. | Whole manuscript |
| **•Some methodological details are insufficient: the phrase “phase 2 of baseline measurements” is ambiguous; it should clearly indicate whether this was a second wave of data collection or a subsample of a longitudinal cohort.** | Thank you for the comment. We have clarified the timeline by explicitly stating that Stage 2 represents the second wave of baseline data collection. | Lines 104-105 |
| **•Definitions of cognitive impairment (based on impairment in ≥1 of 3 tests) may overestimate prevalence; justification or sensitivity analysis using stricter criteria is needed.** | Thank you for the comment.  To maximize sensitivity for case inclusion, the study was informed by the established definitions and consensus criteria of all three assessment tools. |  |
| **•Despite reporting significant associations, the biological and clinical relevance of observed odds ratios (close to 1.0) is minimal and should be interpreted cautiously.** | Thank you for your valuable comment. We have added this point to our limitation section, and we also reviewed the entire manuscript to ensure that any statistically significant findings with minimal clinical relevance were interpreted cautiously and non-significant ones were not reported in the text. | Lines 321-326  Lines 65-67 |
| **•The manuscript states that covariates were selected using backward stepwise regression, which can lead to unstable models and overfitting. Covariate inclusion should be theory-driven or justified by prior evidence.** | Thank you and respect:  In this analysis, variables identified in previous studies as potential confounders for cognitive impairment were first extracted. Subsequently, all of these variables were included in the logistic regression model. Variables with a p-value greater than 0.2 were then removed through a backward elimination process, allowing only the significant variables to remain in the final model. In the next step, the adjusted logistic regression model was developed based on these key variables | Lines 214-218 |
| **•The paper does not describe how missing data were handled. State whether listwise deletion, imputation, or other methods were used.** | Thank you for the comment.  Edited.  The listwise deletion method was used to handle missing data. | Lines 124-129 |
| **•No mention of multicollinearity testing, goodness-of-fit (e.g., Hosmer–Lemeshow test), or sensitivity analyses is provided.** | Thank you for the comment.  Edited. | Tables 3,4,5 |
| •**Effect sizes and confidence intervals are reported but should be discussed in terms of practical importance.** | We appreciate the reviewer's suggestion. We have revised the discussion section to interpret the effect sizes in terms of their modest practical importance, emphasizing the need for additional risk factors and further research to assess clinical relevance. | Lines 321-326  Lines 365-367 |
| **•The decision to stratify by sex is reasonable, but the rationale for doing so should be stated explicitly and, ideally, supported by a statistical test for interaction.** | Thank you and respect  given the significant interactions observed between gender and cognitive impairment with each of the lipid profile components, and considering the potential role of sex as an effect moderator in the association between lipid profile and psychological/cognitive impairment, all analyses were stratified by sex. |  |
| **•The manuscript is understandable, but several sentences are long, redundant, or grammatically inconsistent.** | Thank you. We have carefully checked the entire manuscript and edited long sentences into shorter ones where possible to improve readability. Please let us know if you identify any remaining areas that need further adjustment. | Whole manuscript |
| **•Minor typographical errors occur (“demantia,” “measuremnts”).** | Thank you. Edited. | Line 152- Line 175 |
| **•Paragraphs in the Discussion are overly descriptive; the narrative should be condensed and more analytical.** | Thank you for the valuable feedback. We have condensed overlay data, and added deeper interpretation of the findings, comparisons with previous studies. | Lines 276-286 Lines 352-361 |
| **•Ensure consistent formatting of tables, units (mmol/L vs mg/dL), and reference style. A professional English-language edit is recommended before resubmission.** | We have thoroughly reviewed and edited the manuscript. All values are now uniformly reported in their standard format, with conversion factors added to the Methods section. Table 1 presents demographic characteristics, Table 2 reports all measures in their standard units, and Tables 3–5 present odds ratios with 95% confidence intervals. | Lines 186-203 Tables 1-5 |
|  | | |
| **1.Clarify the study design and population. Clearly describe what “phase 2 of baseline” refers to and specify inclusion/exclusion criteria.** | Thank you for the comment. We have clarified the timeline by explicitly stating that Stage 2 represents the second wave of baseline data collection. | Lines 104-113 |
| **2.Refine the definition of cognitive impairment. Provide justification for your threshold (impairment in ≥1 test) and test an alternative stricter definition in sensitivity analysis.** | Thank you for your feedback.  First, the definition of cognitive impairment was applied separately to each diagnostic tool.  Then, to maximize sensitivity and ensure inclusion of all potential cases of cognitive impairment, a consensus criterion was used that spanned all three assessment tools. |  |
| **3.Re-examine model specification. Replace stepwise regression with theory-driven models or demonstrate that results are robust to different covariate sets.** | Thank you for the comment:  In this analysis, variables identified in previous studies as potential confounders for cognitive impairment were first extracted. Subsequently, all of these variables were included in the logistic regression model. Variables with a p-value greater than 0.2 were then removed through a backward elimination process, allowing only the significant variables to remain in the final model. In the next step, the adjusted logistic regression model was developed based on these key variables | Lines 214-218 |
| **4.Address data handling. Explain how missing values were treated and whether multicollinearity was assessed.** | Thank you for the comment.  Edited  The listwise deletion method was used to handle missing data. | Lines 124-129 |
| **5.Add regression diagnostics. Include measures such as pseudo-R² and goodness-of-fit statistics.** | Thank you for the comment.  Edited. | Tables 3,4,5 |
| **6.Report and interpret effect sizes more cautiously. ORs near 1.0 may be statistically significant but clinically negligible.** | Thank you for your valuable comment. We have added this point to our limitation section, and we also reviewed the entire manuscript to ensure that any statistically significant findings with minimal clinical relevance were interpreted cautiously and non-significant ones were not reported in the text. | Lines 321-326  Lines 367-367 |
| **7.Revise discussion and conclusion. Avoid causal language; discuss mechanisms and confounders (sex hormones, fat distribution, inflammation).** | We have revised the discussion and conclusion to avoid causal language while adding more detail on potential mechanisms and confounders. | Lines 276-286 Lines 347-356 |
| **8.Ensure open data compliance. Deposit anonymized dataset in a public repository and update the Data Availability Statement.** | We have updated the Data Availability Statement to indicate that the anonymized dataset has been deposited in a public repository for open data compliance. | Lines 391-396 |
| **9.Language and formatting. Shorten repetitive sections, standardize tables and figures, and fix minor English errors.** | Thank you for the comment. We have thoroughly reviewed and edited the manuscript. All values are now uniformly reported in their standard format, with conversion factors added to the Methods section. | Whole manuscript |
|  | | |
| **Lines 1–3: “The Association between Novel Anthropometric Indices and Cognitive Function in Iranian Older Adults…” Comment: The title accurately reflects the topic but is lengthy. A concise version such as “****Novel Anthropometric Indices and Cognitive Function among Iranian Older Adults” would improve clarity and alignment with PLOS ONE’s concise title policy.** | We really appreciate your comment. The title is edited, | Lines 1-3 |
| **Lines 4–22: Comment: Multiple affiliations are clearly presented; however, ensure consistent formatting of institutional units (e.g., use “****Tehran University of Medical Sciences, Tehran, Iran” uniformly).** | Thanks. Rechecked. | Lines 4-22 |
| **Abstract** | | |
| **Lines 38–41: The background succinctly introduces TyG, VAI, and LAP, but it would benefit from a stronger rationale explaining why these indices might predict cognitive impairment (e.g., connection to insulin resistance mechanisms).** | Thank you for your comment. Added. | Lines 40-43 |
| **Lines 43–49 – Methods: Specify that logistic regression was sex-stratified a priori to justify separate male/female analyses. Also indicate that confounders were selected via backward stepwise regression.** | Thanks. Edited. | Lines 50-53 |
| **Lines 50–56 – Results: Results are clearly stated but slightly overloaded with numeric detail. Report only the key significant results with directionality; move others to the Results section. Include the total cognitive impairment prevalence (63.9%) only once.** | Some of the less essential data has been removed. | Lines 55-59 |
| **Lines 57–60 – Conclusion: Avoid causal language (“highlight the interplay”) and rephrase to reflect association: “These results suggest complex, sex-specific associations between metabolic indices and cognition.”** | Thank you for your precision. Edited. | Line 62 |
| ****Add the study design (“cross-sectional”) explicitly in the first sentence of the Abstract.** | Added. | Line 38 |
| **Introduction** | | |
| **Lines 69–71: Strong epidemiological framing. Add a recent Iranian study (within the last 3 years) to justify local context.** | Thank you for the suggestion. The sentence has been edited and a recent reference has been added. | Lines 73-74 |
| **Lines 75–92: Excellent description of insulin resistance and novel indices. However, several sentences could be condensed to reduce redundancy (e.g., references 11–17 repeat the mechanistic explanation).** | Thank you for the suggestion. Some of the sentences have been condensed. | Lines 85-87 Lines 90-92 |
| **Lines 93–97: The final paragraph should explicitly define the gap in prior literature—few studies examining TyG, VAI, and LAP together in non-diabetic older adults—and restate the novelty more assertively.** | Thank you for the comment. The paragraph has been revised to clearly define the literature gap and emphasize the novelty of our study. | Lines 95-101 |
| **Study Design** | | |
| **•Clarify timeline: The manuscript says “phase 2 of baseline” measurements”—this phrasing is confusing. Specify if this is the second data collection wave.** | Thank you for the comment. We have clarified the timeline by explicitly stating that Stage 2 represents the second wave of baseline data collection. | Lines 104-105 |
| **•Mention ethical approval and consent here briefly, then expand later.** | Thank you for mentioning that. Added. | Lines 118-120 |
| **•State explicitly that the analysis was conducted in 2025 and that no identifiable data were accessed.** | Thanks. We have explicitly stated that the analysis was conducted in 2025 and that only de-identified data were used. | Lines 122-124 |
| **Data Collection** | | |
| **•Sentence structure unclear (“Data collection included age, sex, income, and level of education, which were gathered…”). Revise for grammatical accuracy.** | We really appreciate your precision. Edited. | Lines 131-132 |
| **•Define how income was categorized.** | Income was collected through direct survey questions and was not used as a standalone categorical variable; instead, it was incorporated as a component of the socioeconomic status index derived using principal component analysis (PCA). | Lines 131-132 |
| **•The PCA-based socio-economic index is mentioned but without variance explained; report the percentage of variance accounted for by the first principal component.** | We have now reported the variance explained by the first principal component; it accounted for 52.0% of the total variance. | 134-137 |
| **•Include the name of the validated questionnaire used (e.g., Global Physical Activity Questionnaire, IPAQ, etc.).** | Thank you. We clarified that we used a validated physical activity questionnaire based on metabolic equivalents, which has been validated in older adults and in its Farsi version, and we added the protocol reference as suggested. | Lines 140-144 |
| **•The MET categories differ from WHO thresholds—justify the classification system or cite a local validation. (Please give the ration or reference)** | We appreciate the comment. The MET categories used in our study follow the physical activity level (PAL) thresholds established by the Institute of Medicine, which are widely applied in epidemiologic and nutrition research. These thresholds differ from WHO classifications but have been validated for estimating habitual activity based on MET-derived PAL ranges. We have added the supporting reference (now cited as reference 27) to justify the classification system. | Reference 27 |
| **Cognitive Tests** | | |
| **•Excellent that three instruments were used. However, the combination rule (“impairment in one or more tests”) could inflate prevalence; justify this decision.** | Thank you for your feedback.  First, the definition of cognitive impairment was applied separately to each diagnostic tool.  Then, to maximize sensitivity and ensure inclusion of all potential cases of cognitive impairment, a consensus criterion was used that spanned all three assessment tools. |  |
| **•Report Cronbach’s α, or internal reliability, for each translated test in the current sample.** | We appreciate the reviewer’s comment. For each translated test, we have now added a brief summary of the available psychometric evidence from their Persian validation studies,including the reported validity and reliability metrics, and have cited the corresponding references to direct readers to the full methodological details. | Lines 153-155  Lines 160-63  Lines 169-170  References 32,34,36 |
| •**Replace “demantia” (typo, line 140) with “dementia.”** | Thank you. Edited. | Line 151 |
| **Physical and Laboratory Measures** | | |
| •**Add the year of calibration or QA/QC procedure for lab assays.** | We have added the year of instrument calibration (2015) and described the internal and external QA/QC procedures to clarify the reliability of laboratory measurements. | Lines 186-187 |
| **•Minor typographical error: “measuremnts” → “measurements.”** | Thanks, edited. | Line 180 |
| **•Define units consistently (e.g., mmol/L or mg/dL, but not both in formulae).** | Thank you. All measures are now based mmol/l with conversion factors added. | Line 202 |
| **Novel Anthropometric Indices** | | |
| •**Formulas are accurate. For transparency, provide conversion factors between mg/dL and mmol/L where applicable.** | Thank you for the suggestion. It’s done. | Lines 191-192 |
| •**Clarify whether lipid and glucose measures were log-transformed before inclusion in models.** | Lipid and glucose measures were not logarithmically transformed before inclusion in the models. |  |
| **Statistical Analysis** | | |
| **Add model diagnostics (e.g., Hosmer–Lemeshow test, multicollinearity check).** | Thanks. Edited as you have suggested | Tables 3,4,5 |
| **Report how missing data were handled (listwise deletion, imputation?).** | Thank you for the comment.  The listwise deletion method was used to handle missing data. | Lines 124-129 |
| **Avoid “backward stepwise”; instead, use theoretical or evidence-based selection to avoid model overfitting.** | Thank you and respect:  In this analysis, variables identified in previous studies as potential confounders for cognitive impairment were first extracted. Subsequently, all of these variables were included in the logistic regression model. Variables with a p-value greater than 0.2 were then removed through a backward elimination process, allowing only the significant variables to remain in the final model. In the next step, the adjusted logistic regression model was developed based on these key variables | Lines 214-218 |
| **State the statistical power or effect size detectable given N=2,426.** | Thank you for highlighting these important points.  Based on the chosen cognitive impairment assessment protocol, an a priori power analysis confirmed that the average statistical power for all analyses—including each test individually and their composite score—exceeded the 80% threshold. |  |
| **Table 1** | | |
| **•Formatting issue: inconsistent column spacing and misaligned p-values.** | We checked and corrected the spacing as possible. | Table 1 and 2 |
| •**Many p-values are reported without correction for multiple testing; consider FDR adjustment.** | Thank you for highlighting these important points.  The false discovery rate (FDR) is a commonly used approach for controlling the Type I error rate in the context of multiple hypothesis testing. In the present study, binary logistic regression was employed to examine the associations of interest. However, given the exploratory nature of this analysis and its primary objective of predicting outcomes, FDR correction was not applied to the reported p-values |  |
| **•Provide units in table headers (e.g., “BMI (kg/m²)”).** | All units are provided in the variables section. | Table 1 and 2 |
| **•The table is overcrowded; consider splitting demographic and biochemical variables** | Nice suggestion, it is done. | Table 1 and 2 |
| **•The text correctly describes significant group differences but overuses repetition. Condense to highlight major findings (e.g., higher prevalence of impairment in females, p < 0.001).** | Thanks. Done. | Lines 230-231 |
| **Results - continued** | | |
| **•Report model goodness-of-fit or pseudo-R² for logistic regressions.** | Thanks. Edited as you have suggested | Table 3,4,5 |
| •**Use consistent precision (two decimals for OR, two for CI limits**). | Thank you for mentioning that. It’s done. | Lines 252-265 |
| •**Clarify whether sex-stratified analyses were exploratory or planned.** | Thank you and respect  given the significant interactions observed between gender and cognitive impairment with each of the lipid profile components, and considering the potential role of sex as an effect moderator in the association between lipid profile and psychological/cognitive impairment, all analyses were stratified by sex. |  |
| **Table 3- 5** | | |
| **•Tables are detailed but overly complex; emphasize key associations in text rather than reproducing all.** | Thanks. We decided to remove some of non-significant associations from the text. | Lines 252-45 |
| **•Clearly label adjusted covariates in each legend.** | Rechecked and edited. | Tables 3-5 |
| **•Highlight significant findings (e.g., p < 0.05) using bold or footnotes rather than asterisks scattered inconsistently.** | Nice suggestion. Added. | Tables 3-5 |
| **•Verify that confidence intervals match p-values; some do not align (e.g., p=0.4 but CI excludes 1).** | Thank you for your precision. We rechecked and edited the numbers. | Tables 3-5 |
| **Discussion** | | |
| **Summary: Concise and appropriate; however, avoid restating numeric results. Instead, emphasize direction** | Revised to avoid repeating numerical results and to emphasize the direction of associations as requested | Lines 273-275 |
| **TyG: Well contextualized. Consider including a physiological rationale for lack of association (e.g., TyG reflects peripheral IR, not central nervous system IR). Current explanation is accurate but could be tightened.** | Thank you; we added a clearer physiological rationale noting that TyG reflects peripheral IR, whereas HOMA-IR captures more systemic insulin signaling with greater relevance to brain function. | Lines 302-304 |
| **VAI: Balanced, but the section mixes discussion of literature with results. Suggest splitting into “Comparison with prior studies” and “Potential mechanisms.” Add a hypothesis why associations differ by sex (e.g., estrogenic modulation, body fat distribution).** | We split the VAI section and added evidence explaining possible sex-related differences in another section. | Lines 281-286 |
| **LAP: Good synthesis, but a brief comment on potential multicollinearity between indices (VAI includes BMI and TG, which also feed into LAP) would enhance rigor.** | Thank you; we added a brief note on potential multicollinearity between VAI and LAP and suggested approaches to address it. | Lies 337-341 |
| **Integrative interpretation: Excellent conceptual framing; could be improved by mentioning inflammation, oxidative stress, or adipokine dysregulation as shared mechanisms.** | Thanks, added. | Lines 344-345 |
| **Limitations: Comprehensive. You may also add: “Residual confounding from unmeasured variables such as diet quality and APOE genotype cannot be excluded.”** | Great observation! This limitation is also included, | Lines 367-370 |
| **Conclusion** | | |
| **Sound and cautious, but slightly repetitive. Revise to two sentences: “In this large cross-sectional study of Iranian older adults, novel anthropometric indices (TyG, LAP, VAI) were not consistently associated with cognitive impairment. Observed sex- and test-specific differences suggest the need for longitudinal studies to clarify causal pathways.”** | Thanks. Edited as you have suggested. | Lines 372-375 |
| **References** | | |
| **•Ensure consistency in reference formatting. •Add 3–4 recent studies (2023–2025) to strengthen currency. https://doi.org/10.1007/s40200-024-01404-8 https://doi.org/10.1002/ncp.11273 https://doi.org/10.1016/j.puhe.2025.01.040 •Verify numbering sequence.** | Thank you for the comment. We have rechecked the citations, ensured consistent formatting, and added more recent studies from 2023–2025, including several relevant studies conducted in Iran. | References – 4-6 |

Reviewer 3:

| **Comments** | **Responses** | **Line number** |
| --- | --- | --- |
| **1. It is essential to provide a separate table that details the all basic characteristics and variables of participants, comparing men and women without subgrouping while reporting the P-values.** | Thanks. Edited as you have suggested | Table 1,2  Lines 232-233  Lines 243-244 |
| **2. A thorough discussion of the advantages and limitations of the FAST, Mini-Cog, and CFT, as above mentioned, should be integrated into the discussion section. Additionally, consider addressing the potential influence of confounding factors, such as educational status, functional abilities, and physical health, which may differ by gender.** | Thank you for highlighting these important points. We have now incorporated the limitations of each cognitive assessment tool into the limitation section. Moreover, the section addressing sex-related differences and their potential influence on the observed associations has been added and integrated into the relevant part of the discussion. | Lines 276-286 |
| **3. Although this study did not measure other non-insulin-based indices of insulin resistance (IR) such as HOMA-IR and TG/HDL, explaining the results mainly through IR mechanisms, which are a vital component of the discussion section, indicates a strong need to revise the discussion section to focus more on the factual gender-specific differences in participant performance in tests that evaluate cognitive impairment.** | Thank you for this important comment. We have revised the discussion to place greater emphasis on the gender-specific differences observed in cognitive test performance and have added more detailed explanations in the relevant paragraphs. The discussion now more clearly distinguishes these empirical findings from broader insulin-resistance–related mechanisms. | Lines 321-330  Lines 308-310 |
| **4. I recommend including a recruitment flow chart that depicts the percentage of excluded participants at each stage, especially the proportion of missing laboratory values.** | Thank you for highlighting these important points.  Edited.  In this study, a total of 2,426 participants were included. In the descriptive analysis, missing data were observed for several independent variables: education, socioeconomic index, and current smoking status (2 cases), blood pressure–lowering medication use (1,347 cases), and glucose-lowering medication use (713 cases). Importantly, there were no missing data for the primary variables of interest, namely lipid profile and cognitive impairment status | Lines 124-129 |
| **5. To enhance clarity, please rephrase “co-association” in line 304**. | Thanks. Edited. | Lines 344-345 |
